# Pediatric Lower Gastrointestinal Bleeding: Endoscopic and Histopathologic Spectrum in a 16-Year Tertiary-Center Cohort

**DOI:** 10.3390/biomedicines14071645

**Published:** 2026-07-21

**Authors:** Kamile Merve Bircan, Abdulkerim Elmas, Mustafa Akçam

**Affiliations:** Division of Pediatric Gastroenterology, Hepatology and Nutrition, Department of Pediatrics, Faculty of Medicine, Suleyman Demirel University, Isparta 32260, Türkiye; akelmas@gmail.com (A.E.); makcam32@gmail.com (M.A.)

**Keywords:** gastrointestinal hemorrhage, colonoscopy, child, inflammatory bowel diseases, intestinal polyps

## Abstract

**Objectives**: Pediatric lower gastrointestinal bleeding encompasses a broad and age-dependent spectrum of conditions, ranging from benign anorectal disorders to inflammatory bowel disease, colorectal polyps, and less common structural or vascular lesions. Despite advances in diagnostic evaluation, contemporary pediatric data on the distribution of underlying diagnoses, particularly according to age and sex, remain limited. This study aimed to evaluate the endoscopic and histopathologic spectrum of pediatric lower gastrointestinal bleeding and to examine age- and sex-related diagnostic patterns. **Methods**: This retrospective single-center study included children aged 0–18 years who presented with overt lower gastrointestinal bleeding and underwent colonoscopic evaluation at a tertiary pediatric gastroenterology center between May 2010 and May 2026. Demographic data, colonoscopic and histopathologic findings, final diagnoses, inflammatory bowel disease subtypes, and polyp histopathology were reviewed. Patients were categorized into four age groups: 0–3, 4–6, 7–12, and 13–18 years. **Results**: A total of 268 children were included. The mean age was 11.4 ± 4.9 years, and 130 patients were female (48.5%). The most common diagnosis was inflammatory bowel disease, identified in 79 patients (29.5%), including 62 with ulcerative colitis and 17 with Crohn’s disease. Normal colonoscopic and histopathologic findings were observed in 65 patients (24.3%), anal fissure in 63 (23.5%), and polypoid lesions in 30 (11.2%). Juvenile polyp was the most common polyp subtype. Diagnostic distribution differed significantly across age groups (*p* < 0.001). Inflammatory bowel disease was more frequent among adolescents, whereas polypoid lesions were most common in children aged 4–6 years. Exploratory sex-based analyses suggested a female predominance in ulcerative colitis and a male predominance in Crohn’s disease and polypoid lesions. **Conclusions**: In children undergoing colonoscopic evaluation for lower gastrointestinal bleeding, inflammatory bowel disease, normal findings, anal fissure, and polypoid lesions were the leading diagnostic categories. Age- and sex-related patterns may help guide clinical evaluation and interpretation of colonoscopic and histopathologic findings.

## 1. Introduction

Lower gastrointestinal bleeding (LGIB) is a common clinical presentation in childhood and may present as hematochezia, visible rectal bleeding, or blood mixed with or coating the stool. Gastrointestinal bleeding has been reported to affect approximately 6.4% of children; however, robust population-based incidence data specifically for pediatric lower gastrointestinal bleeding remain limited. The reported frequency and etiologic spectrum of LGIB vary according to age, geographical region, clinical setting, referral patterns, and patient selection. Consequently, available data are largely derived from selected clinical cohorts and may not accurately reflect the true population-based incidence of pediatric LGIB [1]. The underlying etiology varies considerably according to age, bleeding pattern, clinical presentation, and referral setting. In children, lower gastrointestinal bleeding may result from benign and self-limited conditions such as anal fissure or allergic/eosinophilic proctocolitis, but it may also be the first manifestation of inflammatory bowel disease, colorectal polyps, infectious or nonspecific colitis, vascular lesions, or, rarely, neoplastic conditions [1,2].

The diagnostic approach to pediatric lower gastrointestinal bleeding should be guided by the child’s age, hemodynamic status, amount and duration of bleeding, stool characteristics, bowel habits, abdominal pain, diarrhea, constipation, weight loss, growth failure, anemia, inflammatory markers, and perianal examination findings. National pediatric gastroenterology recommendations also emphasize the importance of a systematic age-based and clinically oriented approach in the evaluation of gastrointestinal bleeding in children [3].

Previous pediatric studies have reported variable etiological distributions for lower gastrointestinal bleeding. Anal fissure, juvenile polyps, infectious or nonspecific colitis, allergic/eosinophilic proctocolitis, and inflammatory bowel disease are among the most frequently reported causes; however, the predominance of each diagnosis differs across studies [4,5,6]. These differences may be related to patient age, indications for colonoscopy, timing of referral, regional characteristics, and whether the study population represents emergency department admissions, outpatient evaluations, or children selected for colonoscopic assessment.

Colonoscopy is a highly valuable diagnostic modality in children with persistent or unexplained LGIB. Endoscopic evaluation allows the detection of mucosal inflammation, polypoid lesions, vascular abnormalities, and rare pathological conditions. In addition, procedures such as biopsy and polypectomy enable diagnosis and treatment to be combined within the same session. The European Society of Gastrointestinal Endoscopy (ESGE) and the European Society for Paediatric Gastroenterology, Hepatology and Nutrition (ESPGHAN) pediatric endoscopy guidelines state that colonoscopy in children with LGIB should be planned according to the clinical condition and appropriate indications [7,8].

Age is one of the major determinants of the differential diagnosis in pediatric lower gastrointestinal bleeding. In infants and young children, allergic/eosinophilic proctocolitis, nonspecific colitis, infectious colitis, and anal fissure are more prominent diagnostic considerations. In preschool and school-aged children, colorectal polyps, particularly juvenile polyps, are an important cause of painless or recurrent rectal bleeding. In older children and adolescents, inflammatory bowel disease becomes increasingly important, especially in the presence of chronic bloody diarrhea, abdominal pain, weight loss, growth impairment, anemia, or elevated inflammatory markers [9,10,11,12].

Although several pediatric series have evaluated the causes of lower gastrointestinal bleeding, data focusing specifically on age- and sex-related differences in colonoscopic and histopathologic diagnoses remain limited. Understanding these patterns may help clinicians prioritize differential diagnoses and interpret colonoscopic findings more effectively in children presenting with lower gastrointestinal bleeding.

Therefore, this study aimed to evaluate the distribution of colonoscopic and histopathologic diagnoses among children who underwent colonoscopic evaluation for lower gastrointestinal bleeding in a tertiary pediatric gastroenterology center between May 2010 and May 2026, and to examine the variation in these diagnoses according to age group and sex.

## 2. Methods

### 2.1. Study Design and Participants

This retrospective, single-center study was conducted at the Pediatric Gastroenterology, Hepatology and Nutrition Clinic of Suleyman Demirel University. The medical records of all pediatric patients who underwent colonoscopic evaluation between May 2010 and May 2026 were retrospectively screened. Patients who underwent colonoscopy for overt lower gastrointestinal bleeding were then assessed for eligibility according to the predefined inclusion and exclusion criteria.

For the purposes of this study, lower gastrointestinal bleeding was defined as hematochezia, visible fresh rectal bleeding, or visible blood mixed with or coating the stool. Patients were included if they were aged 0–18 years, presented with overt lower gastrointestinal bleeding, underwent colonoscopic evaluation during the study period, and had available colonoscopic and/or histopathologic diagnostic data.

**Figure 1:**
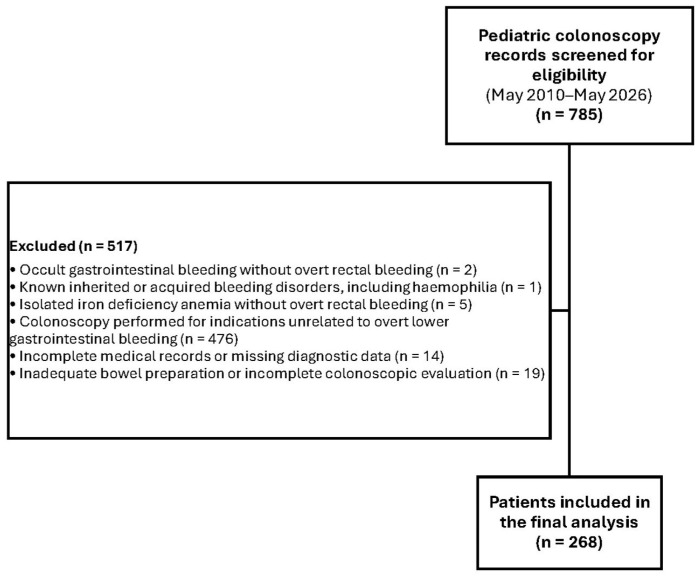
Flow diagram of study population selection.

Patients were included if they were aged 0–18 years, presented with overt lower gastrointestinal bleeding, underwent colonoscopic evaluation during the study period, and had available diagnostic data. Patients evaluated solely for occult gastrointestinal bleeding, isolated iron deficiency anemia without overt rectal bleeding, or indications unrelated to overt lower gastrointestinal bleeding were excluded. Additional exclusion criteria were known inherited or acquired bleeding disorders, including haemophilia, incomplete medical records or missing diagnostic data, inadequate bowel preparation, and incomplete colonoscopic evaluation. The patient selection process is presented in Figure 1.

Demographic data, including age and sex, were recorded. Patients were categorized into four age groups: 0–3 years, 4–6 years, 7–12 years, and 13–18 years. These age groups were selected to reflect clinically relevant developmental stages and age-related variation in the differential diagnosis of lower gastrointestinal bleeding in children.

### 2.2. Colonoscopic and Histopathologic Evaluation

All colonoscopic examinations were performed by pediatric gastroenterologists using Fujinon 4450 series video endoscopes (Fujifilm Corporation, Tokyo, Japan) under sedation with midazolam and ketamine according to institutional practice, a combination that has been previously described and evaluated for pediatric gastrointestinal endoscopic procedures [13]. Patients with inadequate bowel preparation were excluded from the study. Cecal intubation and terminal ileal intubation status were recorded when available. Mucosal biopsies were routinely obtained, including from endoscopically normal-appearing mucosa when clinically appropriate, and histopathologic diagnoses were based on the original pathology reports issued by the Department of Pathology.

Colonoscopic findings, histopathologic results, number of procedures, repeat colonoscopies, polypectomy or biopsy findings, and final clinical diagnoses were reviewed. Each patient was assigned to a single final diagnostic category. In patients with more than one potential finding, classification was based on the final clinical diagnosis documented by the treating pediatric gastroenterologist after review of all available clinical, endoscopic, histopathologic, radiological, and follow-up data. Therefore, analyses were performed at the patient level rather than the procedure level. Repeat colonoscopies performed for disease monitoring, treatment response evaluation, or surveillance were not analyzed as separate observations.

Diagnostic categories included inflammatory bowel disease, anal fissure, normal colonoscopic and histopathologic findings, polypoid lesion, nonspecific colitis, eosinophilic colitis, IgA vasculitis-associated colitis, rectal ulcer, hemangioma, neuroendocrine tumor, and other rare lesions when present. Inflammatory bowel disease was further classified as ulcerative colitis or Crohn’s disease according to the final recorded diagnosis. However, ulcerative colitis and Crohn’s disease were evaluated as subgroups of inflammatory bowel disease and were not counted as separate diagnostic categories in the overall diagnostic distribution.

The diagnosis of inflammatory bowel disease was based on clinical, endoscopic, histopathologic, radiological, and follow-up findings documented in the medical records, in accordance with contemporary pediatric gastroenterology criteria [9]. Infectious etiologies were excluded when clinically indicated. Upper gastrointestinal endoscopy and small-bowel imaging, including magnetic resonance enterography, were performed in selected patients when Crohn’s disease was suspected or when additional evaluation was considered necessary.

In patients with polypoid lesions, histopathologic subtypes were recorded as juvenile polyp, adenomatous polyp, inflammatory polyp, hyperplastic polyp, and unclassified polyp. Histological classification was based on pathological examination of biopsy or polypectomy specimens and not solely on endoscopic appearance.

### 2.3. Statistical Analysis

Categorical variables were expressed as numbers and percentages. Continuous variables were expressed as mean ± standard deviation or median with interquartile range, according to data distribution. The distribution of diagnostic categories across age groups was evaluated using Pearson’s chi-square test. When expected cell counts were low, Fisher’s exact test or Monte Carlo exact significance values were used, as appropriate. For sex-based comparisons, each diagnostic category was compared with all other patients. A *p* value of <0.05 was considered statistically significant. Statistical analyses were performed using IBM SPSS Statistics for Windows, version 27.0 (IBM Corp., Armonk, NY, USA).

### 2.4. Ethical Considerations

Patient data were anonymized before analysis, and confidentiality was maintained throughout the study. The manuscript was prepared in accordance with the STROBE guidelines. The study was conducted in accordance with the principles of the Declaration of Helsinki and was approved (approval no: 122/1; date: 5 June 2026) by the Ethics Committee of Suleyman Demirel University Faculty of Medicine.

## 3. Results

During the study period, 268 children who underwent colonoscopic evaluation for lower gastrointestinal bleeding between May 2010 and May 2026 were included in the final analysis.

Of the 268 patients, 138 (51.5%) were male and 130 (48.5%) were female. The mean age was 11.4 ± 4.9 years, and the median age was 12 years (IQR: 7–16 years). The largest age group consisted of adolescents aged 13–18 years (*n* = 127, 47.4%), followed by children aged 7–12 years (*n* = 84, 31.3%), 4–6 years (*n* = 39, 14.6%), and 0–3 years (*n* = 18, 6.7%) (Table 1).

The most common diagnostic category was inflammatory bowel disease, identified in 79 patients (29.5%), followed by normal colonoscopic and histopathologic findings (24.3%), anal fissure (23.5%), and polypoid lesions (11.2%). Less frequent diagnoses included nonspecific colitis, eosinophilic colitis, IgA vasculitis-associated colitis, rectal ulcer, hemangioma, and neuroendocrine tumor, as detailed in Table 2.

Hemangioma was observed in a 7-year-old male patient as a mucosal lesion measuring 1 × 1 cm in the rectosigmoid region. The lesion was considered a potential source of bleeding and was managed conservatively because the patient remained clinically stable during follow-up. The neuroendocrine tumor was detected in a 6-year-old male patient as a Grade 1 lesion measuring 1 × 1.5 cm and located in the rectum. Following diagnosis, the patient was referred to the pediatric hematology and oncology department for further evaluation and management (Table 2).

Among patients with polyps (*n* = 30), the most common histopathologic subtype was juvenile polyp (*n* = 16, 53.3%). This was followed by adenomatous polyp (*n* = 6, 20%), inflammatory polyp (*n* = 4, 13.3%), and hyperplastic polyp (*n* = 3, 10%). In one patient (3.3%), the polyp type could not be classified. Polypectomy was performed in 26 patients. In the remaining four patients, the lesions were considered too small for polypectomy and tissue samples were obtained by biopsy. All polypoid lesions underwent histopathologic evaluation.

The distribution of diagnoses differed significantly across age groups (overall *p* < 0.001). Inflammatory bowel disease increased with age and was most frequent among adolescents aged 13–18 years, whereas polypoid lesions were most frequent in children aged 4–6 years. Nonspecific colitis and eosinophilic colitis were more prominent in the youngest age group. The complete age-specific distribution of diagnoses is presented in Table 3.

When the distribution of diagnoses was evaluated according to sex, inflammatory bowel disease was diagnosed in 46 girls (58.2%) and 33 boys (41.8%), and this difference was statistically significant (*p* = 0.040). Among inflammatory bowel disease subtypes, ulcerative colitis was more frequent in girls, with 42 girls (67.7%) and 20 boys (32.3%) diagnosed with ulcerative colitis (*p* < 0.001). In contrast, Crohn’s disease was more frequent in boys, with 13 boys (76.5%) and 4 girls (23.5%) diagnosed with Crohn’s disease (*p* = 0.033). Among patients with anal fissure, 34 were female (54.0%) and 29 were male (46.0%), with no significant difference between sexes (*p* = 0.321). Polypoid lesions were more frequently observed in boys, with 21 boys (70.0%) and 9 girls (30.0%) diagnosed with polyps (*p* = 0.031).

No significant sex-based differences were observed for nonspecific colitis, eosinophilic colitis, normal colonoscopic and histopathologic findings, or rare diagnoses including IgA vasculitis-associated colitis, rectal ulcer, hemangioma, and neuroendocrine tumor.

Overall, inflammatory bowel disease and ulcerative colitis were more frequently observed in girls, whereas Crohn’s disease and polypoid lesions were more frequently observed in boys. These subgroup findings should be interpreted cautiously because the sex-based analyses were exploratory and were not adjusted for multiple comparisons (Table 4).

## 4. Discussion

In this 16-year retrospective cohort of children who underwent colonoscopic evaluation for lower gastrointestinal bleeding, inflammatory bowel disease was the most common diagnostic category, followed by normal colonoscopic and histopathologic findings, anal fissure, and polypoid lesions. Inflammatory bowel disease accounted for 29.5% of the cohort, whereas normal findings, anal fissure, and polypoid lesions were observed in 24.3%, 23.5%, and 11.2% of patients, respectively. The diagnostic spectrum showed significant age-related variation. Inflammatory bowel disease was most prominent among adolescents, polypoid lesions were most frequent in children aged 4–6 years, and nonspecific colitis and eosinophilic colitis were more common in younger children. These findings underline the importance of interpreting pediatric lower gastrointestinal bleeding within an age-oriented and clinically contextualized framework.

The reported etiologic spectrum of pediatric lower gastrointestinal bleeding varies substantially across studies. In our cohort, inflammatory bowel disease was the leading diagnosis, whereas Paul et al. recently reported anal fissure as the most frequent cause of LGIB in a small South Indian pediatric cohort, followed by proctitis/colitis and polyps [14]. Turkish pediatric colonoscopy and gastrointestinal bleeding series have likewise reported heterogeneous distributions, including inflammatory bowel disease, colorectal polyps, anal fissure, and normal colonoscopic or histopathologic findings [15,16], while international studies have shown varying predominance of anal fissure, juvenile polyps, colitis, or inflammatory bowel disease [1,2,17]. These discrepancies are unlikely to reflect geographic variation alone. Differences in patient age, referral pathways, clinical setting, case definition, threshold for endoscopic evaluation, and the extent of diagnostic work-up may substantially influence the observed distribution of diagnoses. In particular, our cohort consisted exclusively of children selected for colonoscopic evaluation at a tertiary pediatric gastroenterology center, which likely enriched the study population for persistent, recurrent, or clinically concerning bleeding and therefore for conditions such as inflammatory bowel disease. In contrast, cohorts including children assessed in broader clinical settings may contain a greater proportion of benign anorectal causes such as anal fissure. Consequently, comparisons across studies should take differences in case selection and diagnostic methodology into account rather than assuming that the reported frequencies represent true population-level differences.

This selection effect also has important clinical implications. Our findings should not be interpreted as supporting colonoscopy for every child with minor rectal bleeding. Rather, endoscopic evaluation is most informative in selected patients with persistent or recurrent bleeding, alarm features, or suspected mucosal disease. In this context, ileocolonoscopy combined with histopathologic assessment can help distinguish inflammatory bowel disease, polypoid lesions, nonspecific inflammatory changes, and less common structural or vascular lesions [1,7,8].

The age-stratified analysis showed that inflammatory bowel disease increased markedly with age and was most frequent in adolescents. In the 13–18-year age group, inflammatory bowel disease was detected in 42.5% of patients. This finding is consistent with the known epidemiology of pediatric inflammatory bowel disease, which is more commonly diagnosed in school-aged children and adolescents than in infants or preschool-aged children. Rectal bleeding in this age group should therefore prompt careful assessment for accompanying features such as chronic diarrhea, abdominal pain, weight loss, growth impairment, anemia, elevated inflammatory markers, and increased fecal calprotectin when available. Current pediatric inflammatory bowel disease diagnostic criteria emphasize the role of ileocolonoscopy with histopathologic assessment, together with upper gastrointestinal endoscopy and small-bowel imaging when Crohn’s disease is suspected [9]. In our cohort, ulcerative colitis represented the majority of inflammatory bowel disease cases, which is expected in a study population defined by overt lower gastrointestinal bleeding. The marked increase in IBD among adolescents in our cohort may reflect both the age-related epidemiology of pediatric IBD and referral enrichment, as older children with persistent bloody diarrhea and systemic or inflammatory features are more likely to undergo comprehensive endoscopic evaluation. Therefore, the observed age gradient should be interpreted as a combination of underlying disease epidemiology and selection within a tertiary colonoscopy-based cohort rather than as a direct estimate of age-specific population risk.

Anal fissure was one of the leading diagnostic categories and was identified in 23.5% of patients. Anal fissure is a common cause of bright red rectal bleeding in childhood, particularly in association with constipation, hard stools, and painful defecation. Previous studies have reported anal fissure as one of the most frequent causes of pediatric lower gastrointestinal bleeding, especially in non-tertiary and non-colonoscopy-based settings [1,2,18,19]. The relatively high rate of anal fissure in our colonoscopy-assessed cohort reinforces the importance of meticulous perianal examination before proceeding to invasive investigations. However, the presence of an anal fissure should not automatically end the diagnostic evaluation in children with recurrent bleeding, anemia, chronic diarrhea, growth failure, systemic symptoms, or biochemical evidence of inflammation. In such cases, a visible perianal source may coexist with or obscure a more proximal intestinal pathology.

Normal colonoscopic and histopathologic findings were observed in 24.3% of patients, making this the second most common diagnostic category in the updated cohort. The frequency of normal colonoscopic findings in children evaluated for lower gastrointestinal bleeding varies widely in the literature [20,21]. Several factors may explain this variability, including intermittent bleeding, spontaneous resolution of transient infectious or inflammatory changes, bleeding from a perianal source that is not active at the time of examination, prior treatment, bowel preparation effects, and differences in referral thresholds. In our study, normal findings were distributed relatively similarly across age groups. This suggests that a negative colonoscopic and histopathologic evaluation is not restricted to a particular developmental period. From a clinical perspective, subsequent management should be individualized according to the clinical course. In children in whom bleeding has resolved and who have no anemia, growth impairment, systemic symptoms, or other alarm features, careful clinical follow-up may be appropriate. Persistent or recurrent bleeding should prompt reassessment of the bleeding pattern and perianal region and, depending on the clinical context, additional evaluation including complete blood count, iron status, inflammatory markers, stool-based investigations, and further gastrointestinal assessment. Peripheral blood smear examination and more detailed hematologic evaluation may also be considered when abnormalities in blood cell counts or morphology are present. A normal colonoscopic and histopathologic examination should therefore be interpreted as the absence of an identifiable colonic mucosal lesion at the time of assessment rather than as definitive exclusion of all potential causes of gastrointestinal bleeding. Future prospective studies may also investigate whether detailed hematologic phenotyping or blood-based molecular approaches can improve diagnostic stratification in children with otherwise unexplained lower gastrointestinal bleeding. Genomic, proteomic, and epigenomic, including methylomic, approaches may provide novel mechanistic and biomarker information; however, these investigations require prospectively collected biological specimens and were beyond the scope of the present retrospective study.

Polypoid lesions were detected in 11.2% of patients, and juvenile polyps were the most common histopathologic subtype. This finding is consistent with the established role of colorectal polyps as an important cause of painless or recurrent rectal bleeding in childhood [10,11,12]. The highest frequency of polyps was observed in children aged 4–6 years, supporting the typical presentation of juvenile polyps during preschool and early school-age periods. Colonoscopy has a distinct advantage in this context because it allows diagnosis and treatment during the same procedure through polypectomy. In our cohort, polypoid lesions were more frequent in boys. Although this difference reached statistical significance, it should be interpreted cautiously because subgroup analyses were exploratory and were not adjusted for multiple comparisons. Moreover, previous pediatric studies have reported inconsistent findings regarding sex distribution in childhood colorectal polyps, suggesting that local cohort structure and sample size may influence this observation [10,11,12]. Differences in the reported frequency of polyps across studies may also reflect variation in age distribution, referral practices, and whether cohorts include all children with rectal bleeding or only those undergoing complete colonoscopic evaluation.

Nonspecific colitis and eosinophilic colitis were more prominent in younger children. Nonspecific colitis was the most common diagnosis in the 0–3-year age group, and eosinophilic colitis also clustered in early childhood. In infants and young children with bloody stools, the differential diagnosis differs from that in adolescents and includes food protein-induced allergic proctocolitis, eosinophilic proctocolitis, infectious colitis, and transient nonspecific mucosal inflammation [21,22]. This age-related pattern is clinically relevant because inflammatory bowel disease is less common in this age group, whereas benign or self-limited inflammatory conditions are more frequently encountered. Nevertheless, persistent symptoms, poor growth, severe anemia, or atypical endoscopic findings should still prompt broader evaluation, including consideration of very-early-onset inflammatory bowel disease when clinically appropriate.

The sex-based analysis showed that inflammatory bowel disease overall and ulcerative colitis were more frequent in girls, whereas Crohn’s disease and polypoid lesions were more frequent in boys. Sex-related differences in pediatric inflammatory bowel disease have been described in previous studies, but the direction and magnitude of these differences vary according to age, population, disease subtype, and study design [23,24,25]. Therefore, the sex-related findings in our cohort should be interpreted as hypothesis-generating rather than definitive. The female predominance in ulcerative colitis and male predominance in Crohn’s disease may reflect true biological variation, but may also be influenced by sample size, age distribution, referral characteristics, or unmeasured clinical variables. Larger multicenter pediatric cohorts are needed to clarify whether these sex-related patterns are reproducible.

Rare diagnoses in this cohort included IgA vasculitis-associated colitis, rectal ulcer, hemangioma, and neuroendocrine tumor. Although these conditions were infrequent, they are clinically important because they demonstrate the broader diagnostic yield of colonoscopy in selected children with persistent or unexplained lower gastrointestinal bleeding. Vascular lesions, solitary rectal ulcer, inflammatory mucosal lesions, and rare neoplastic conditions should be considered when bleeding is recurrent, unexplained, or associated with anemia, weight loss, or suspicious endoscopic findings. Malignant and premalignant lesions are rare in childhood, but they should not be dismissed entirely in the presence of persistent symptoms or focal lesions. The identification of a rectal neuroendocrine tumor in one patient in our cohort highlights the value of histopathologic confirmation even when rare diagnoses are unexpected [2,19,26].

Although rare causes were infrequent in our cohort, the differential diagnosis of pediatric lower gastrointestinal bleeding should remain broad in selected patients. Foreign body ingestion may occasionally cause mucosal injury and bleeding, particularly in younger children or when sharp objects, button batteries, or magnets are involved [1,2]. In children with persistent or recurrent bleeding in whom colonoscopic and radiologic evaluations fail to identify the source, surgically correctable conditions such as Meckel’s diverticulum should also be considered, and laparoscopy may provide both diagnostic and therapeutic benefit in carefully selected cases [27,28]. Overall management should be guided by bleeding severity, hemodynamic status, and underlying etiology, with endoscopy serving both diagnostic and therapeutic roles when clinically indicated [7,8].

This study has several strengths. It represents a 16-year experience from a tertiary pediatric gastroenterology center and includes a relatively large cohort of children who underwent colonoscopic evaluation for lower gastrointestinal bleeding. The use of final clinical diagnoses supported by colonoscopic and histopathologic data allowed mutually exclusive diagnostic classification at the patient level. In addition, the age- and sex-stratified analyses provide clinically useful information for differential diagnosis and may help clinicians interpret colonoscopic findings according to developmental stage and patient profile.

The study also has limitations. Its retrospective and single-center design limits generalizability. Because the cohort included only children who underwent colonoscopic evaluation in a tertiary referral center, the findings should not be interpreted as the population-based etiologic distribution of pediatric lower gastrointestinal bleeding. Detailed clinical and laboratory variables, including bleeding duration and severity, stool characteristics, constipation, diarrhea, abdominal pain, weight loss, growth parameters, hemoglobin levels, inflammatory markers, fecal calprotectin, stool culture results, medication exposure, family history, and perianal examination findings, were not consistently available and therefore could not be analyzed in a standardized manner. In particular, although family history may be clinically relevant for conditions such as inflammatory bowel disease and colorectal polyps, the incompleteness of retrospective documentation precluded a reliable association analysis and could have introduced information and misclassification bias. Future prospective studies should systematically collect family history and evaluate its relationship with diagnostic patterns in children presenting with lower gastrointestinal bleeding. Infectious etiologies could not be systematically excluded in all patients classified as having nonspecific colitis. In addition, because of the long study period, diagnostic investigations and histopathologic assessments were performed as part of routine clinical practice and were not fully standardized across all patients. Finally, sex-based subgroup findings should be interpreted cautiously because some diagnostic categories included small numbers and multiple-comparison adjustment was not performed.

## 5. Conclusions

In this study, inflammatory bowel disease, normal colonoscopic and histopathologic findings, anal fissure, and polypoid lesions were the leading diagnostic categories among children who underwent colonoscopic evaluation for lower gastrointestinal bleeding. The diagnostic spectrum varied according to age and sex. Inflammatory bowel disease was more frequently observed in adolescents, whereas polypoid lesions were most common in children aged 4–6 years, and nonspecific colitis and eosinophilic colitis were more prominent in younger children. In sex-based analyses, inflammatory bowel disease and ulcerative colitis were more frequent in girls, while Crohn’s disease and polypoid lesions were more frequent in boys. These findings suggest that age, sex, clinical presentation, perianal examination, colonoscopic findings, and histopathologic evaluation should be considered together in the diagnostic assessment of pediatric lower gastrointestinal bleeding.

## Figures and Tables

**Table 1:** Demographic Characteristics of the Study Population (*n* = 268).

Characteristic	Value
Sex, *n* (%)	
Male	138 (51.5)
Female	130 (48.5)
Age, years, mean ± SD	11.4 ± 4.9
Age, years, median (IQR)	12 (7–16)
Age group, years, *n* (%)	
0–3	18 (6.7)
4–6	39 (14.6)
7–12	84 (31.3)
13–18	127 (47.4)

*SD: standard deviation; IQR: interquartile range.*

**Table 2:** Distribution of Colonoscopic and Histopathologic Diagnoses in Children with Lower Gastrointestinal Bleeding.

Diagnoses	*n* (%)
Inflammatory bowel disease	79 (29.5)
Ulcerative colitis *	62 (78.5)
Crohn’s disease *	17 (21.5)
Normal colonoscopic and histopathologic findings	65 (24.3)
Anal fissure	63 (23.5)
Polypoid lesions	30 (11.2)
Nonspecific colitis	19 (7.1)
Eosinophilic colitis	6 (2.2)
IgA vasculitis-associated colitis	2 (0.7)
Rectal ulcer	2 (0.7)
Hemangioma	1 (0.4)
Neuroendocrine tumor	1 (0.4)

* Ulcerative colitis and Crohn’s disease are presented as subtypes of inflammatory bowel disease. Percentages for these subtypes were calculated within the inflammatory bowel disease group and were not included as separate diagnostic categories in the overall diagnostic distribution.

**Table 3:** Distribution of Colonoscopic and Histopathologic Diagnoses According to Age Group.

Diagnosis	0–3 Years *n* (%)	4–6 Years *n* (%)	7–12 Years *n* (%)	13–18 Years *n* (%)	*p*
Inflammatory bowel disease	2 (11.1)	4 (10.3)	19 (22.6)	54 (42.5)	**<0.001**
Ulcerative colitis *	2 (100)	3 (75.0)	15 (78.9)	42 (77.8)	
Crohn’s disease *	0 (0)	1 (25.0)	4 (21.1)	12 (22.2)	
Anal fissure	2 (11.1)	8 (20.5)	23 (27.4)	30 (23.6)	0.530 **^β^**
Polypoid lesions	1 (5.6)	10 (25.6)	12 (14.3)	7 (5.5)	**0.004 ^β^**
Nonspecific colitis	6 (33.3)	3 (7.7)	6 (7.1)	4 (3.1)	**0.001 ^β^**
Eosinophilic colitis	3 (16.7)	2 (5.1)	0 (0)	1 (0.8)	**0.001 ^β^**
IgA vasculitis-associated colitis	0 (0)	0 (0)	1 (1.2)	1 (0.8)	1.000 **^β^**
Rectal ulcer	0 (0)	1 (2.6)	0 (0)	1 (0.8)	0.480 **^β^**
Hemangioma	0 (0)	0 (0)	1 (1.2)	0 (0)	0.527 **^β^**
Neuroendocrine tumor	0 (0)	1 (2.6)	0 (0)	0 (0)	0.213 **^β^**
Normal colonoscopic and histopathologic findings	4 (22.2)	10 (25.6)	22 (26.2)	29 (22.8)	0.946 **^β^**
Total	18	39	84	127	

* Percentages for ulcerative colitis and Crohn’s disease were calculated within the inflammatory bowel disease subgroup. p values indicate comparisons of each diagnostic category across age groups. Pearson’s chi-square test was used when assumptions were met; otherwise, Fisher’s exact test **^β^** *or Monte Carlo exact significance values were used for cells with low expected counts. Bold p values indicate statistical significance.*

**Table 4:** Distribution of Colonoscopic and Histopathologic Diagnoses According to Sex.

Diagnosis	Male *n* (%)	Female *n* (%)	*p* *
Inflammatory bowel disease	33 (41.8)	46 (58.2)	**0.040**
Ulcerative colitis	20 (32.3)	42 (67.7)	**<0.001**
Crohn’s disease	13 (76.5)	4 (23.5)	**0.033**
Anal fissure	29 (46.0)	34 (54.0)	0.321
Polypoid lesions	21 (70.0)	9 (30.0)	**0.031**
Nonspecific colitis	10 (52.6)	9 (47.4)	0.918
Eosinophilic colitis	2 (33.3)	4 (66.7)	0.435 **^β^**
IgA vasculitis-associated colitis	2 (100)	0 (0)	0.499 **^β^**
Rectal ulcer	2 (100)	0 (0)	0.499 **^β^**
Hemangioma	1 (100)	0 (0)	1.000 **^β^**
Neuroendocrine tumor	1 (100)	0 (0)	1.000 **^β^**
Normal colonoscopic and histopathologic findings	37 (56.9)	28 (43.1)	0.314
Total	138	130	

* *p values were calculated by comparing each diagnostic group with all other patients. Pearson’s chi-square test was used when expected cell counts were adequate, whereas Fisher’s exact test* **^β^** *was used when expected cell counts were low. Percentages indicate the sex distribution within each diagnostic category. Bold p values indicate statistical significance.*

## Data Availability

The data presented in this study are not publicly available due to privacy and ethical restrictions. De-identified data may be available from the corresponding author upon reasonable request and with permission from the relevant institutional authorities.

## Abbreviations

The following abbreviations are used in this manuscript:

CD	Crohn’s disease
ESGE	European Society of Gastrointestinal Endoscopy
ESPGHAN	European Society for Paediatric Gastroenterology, Hepatology and Nutrition
IBD	Inflammatory bowel disease
IgA	Immunoglobulin A
IQR	Interquartile range
LGIB	Lower gastrointestinal bleeding
NET	Neuroendocrine tumor
SD	Standard deviation
UC	Ulcerative colitis
